# Evaluating a Natural Language Processing–Driven, AI-Assisted International Classification of Diseases, 10th Revision, Clinical Modification, Coding System for Diagnosis Related Groups in a Real Hospital Environment: Algorithm Development and Validation Study

**DOI:** 10.2196/58278

**Published:** 2024-09-20

**Authors:** Hong-Jie Dai, Chen-Kai Wang, Chien-Chang Chen, Chong-Sin Liou, An-Tai Lu, Chia-Hsin Lai, Bo-Tsz Shain, Cheng-Rong Ke, William Yu Chung Wang, Tatheer Hussain Mir, Mutiara Simanjuntak, Hao-Yun Kao, Ming-Ju Tsai, Vincent S Tseng

**Affiliations:** 1 Intelligent System Lab, College of Electrical Engineering and Computer Science Department of Electrical Engineering National Kaohsiung University of Science and Technology Kaohsiung Taiwan; 2 National Institute of Cancer Research National Health Research Institutes Tainan Taiwan; 3 Center for Big Data Research Kaohsiung Medical University Kaohsiung Taiwan; 4 Department of Computer Science National Yang Ming Chiao Tung University Hsinchu Taiwan; 5 Advanced Technology Laboratory Chunghwa Telecom Laboratories Taoyuan Taiwan; 6 Electromagnetic Sensing Control and AI Computing System Laboratory Department of Electrical Engineering, College of Electrical Engineering and Computer Science National Kaohsiung University of Science and Technology Kaohsiung Taiwan; 7 School of Post-Baccalaureate Medicine Kaohsiung Medical University Kaohsiung Taiwan; 8 Waikato Management School University of Waikato Hamilton New Zealand; 9 Department of Healthcare Administration and Medical Informatics College of Health Sciences Kaohsiung Medical University Kaohsiung Taiwan; 10 Division of Pulmonary and Critical Care Medicine Department of Internal Medicine Kaohsiung Medical University Hospital, Kaohsiung Medical University Kaohsiung Taiwan

**Keywords:** natural language processing, International Classification of Diseases, deep learning, electronic medical record, Taiwan diagnosis related groups

## Abstract

**Background:**

International Classification of Diseases codes are widely used to describe diagnosis information, but manual coding relies heavily on human interpretation, which can be expensive, time consuming, and prone to errors. With the transition from the *International Classification of Diseases, Ninth Revision*, to the *International Classification of Diseases, Tenth Revision* (*ICD-10*), the coding process has become more complex, highlighting the need for automated approaches to enhance coding efficiency and accuracy. Inaccurate coding can result in substantial financial losses for hospitals, and a precise assessment of outcomes generated by a natural language processing (NLP)–driven autocoding system thus assumes a critical role in safeguarding the accuracy of the Taiwan diagnosis related groups (Tw-DRGs).

**Objective:**

This study aims to evaluate the feasibility of applying an *International Classification of Diseases, Tenth Revision, Clinical Modification* (*ICD-10-CM*), autocoding system that can automatically determine diagnoses and codes based on free-text discharge summaries to facilitate the assessment of Tw-DRGs, specifically principal diagnosis and major diagnostic categories (MDCs).

**Methods:**

By using the patient discharge summaries from Kaohsiung Medical University Chung-Ho Memorial Hospital (KMUCHH) from April 2019 to December 2020 as a reference data set we developed artificial intelligence (AI)–assisted *ICD-10-CM* coding systems based on deep learning models. We constructed a web-based user interface for the AI-assisted coding system and deployed the system to the workflow of the certified coding specialists (CCSs) of KMUCHH. The data used for the assessment of Tw-DRGs were manually curated by a CCS with the principal diagnosis and MDC was determined from discharge summaries collected at KMUCHH from February 2023 to April 2023.

**Results:**

Both the reference data set and real hospital data were used to assess performance in determining *ICD-10-CM* coding, principal diagnosis, and MDC for Tw-DRGs. Among all methods, the GPT-2 (OpenAI)-based model achieved the highest *F*_1_-score, 0.667 (*F*_1_-score 0.851 for the top 50 codes), on the KMUCHH test set and a slightly lower *F*_1_-score, 0.621, in real hospital data. Cohen κ evaluation for the agreement of MDC between the models and the CCS revealed that the overall average κ value for GPT-2 (κ=0.714) was approximately 12.2 percentage points higher than that of the hierarchy attention network (κ=0.592). GPT-2 demonstrated superior agreement with the CCS across 6 categories of MDC, with an average κ value of approximately 0.869 (SD 0.033), underscoring the effectiveness of the developed AI-assisted coding system in supporting the work of CCSs.

**Conclusions:**

An NLP-driven AI-assisted coding system can assist CCSs in *ICD-10-CM* coding by offering coding references via a user interface, demonstrating the potential to reduce the manual workload and expedite Tw-DRG assessment. Consistency in performance affirmed the effectiveness of the system in supporting CCSs in *ICD-10-CM* coding and the judgment of Tw-DRGs.

## Introduction

### Background

The International Classification of Diseases (ICD) [1], established by the World Health Organization, is a crucial medical classification system that defines the universe of diseases, disorders, injuries, and other related health conditions. Since its first publication in 1893, the ICD has become a widely adopted standard across various health care facilities and settings globally, providing consistency and accuracy in disease diagnosis and classification. In 1992, the World Health Organization published the *International Classification of Diseases, Tenth Revision* (*ICD-10*), which has since been widely adopted worldwide [2,3]. Many countries extended and customized the *ICD-10* classification system for their country-specific reporting purposes, such as *International Classification of Diseases, Tenth Revision, Clinical Modification* (*ICD-10-CM*) in the United States, *International Classification of Diseases, Tenth Revision, Canadian Modification* in Canada, *International Classification of Diseases, Tenth Revision, German Modification* in Germany, and *International Classification of Diseases, Tenth Revision, Australian Modification* that is followed by Australia as well as 15 other countries including Ireland, Singapore, and Saudi Arabia.

The *ICD-10-CM* is an ICD system that classifies patients according to the type of illness, severity, and the location of the disease that was developed to describe more clinical details with the increasing number of diagnoses and procedural codes applied in payment methodologies [4,5]. As a result, the *ICD-10-CM* coding task has become a crucial element in various fields, such as disease surveillance [6], health services management [7], and clinical research [8]. For the National Health Insurance Administration (NHIA) in Taiwan, *ICD-10-CM* holds immense significance by serving as a standardized coding system for the statistical analysis of disease diagnosis, surgical treatment for patients admitted to hospital, and payment of health insurance. In 2016, the NHIA in Taiwan followed the global trend and transitioned from the International *Classification of Diseases, Ninth Revision, Clinical Modification* (*ICD-9*-*CM*), to *ICD-10-CM*, which expanded the number of codes available. Previously, the *ICD-9*-*CM* contained approximately 14,000 diagnosis codes, while the *ICD-10-CM* had approximately 69,000 diagnosis codes [5]. The NHIA is currently using the 2014 version of *ICD-10-CM*, which consists of approximately 71,900 diagnosis codes. Nowadays, many medical institutions are relying on licensed certified coding specialists (CCSs) to manually assign *ICD-10* codes to inpatients. These coders spend a significant amount of time reviewing various medical materials to accurately diagnose and code each patient’s condition. Due to the complexity of the ICD-10 structure and coding rules, the task of coding is significantly more labor-intensive and time-consuming than *ICD-9*, even when performed by a skilled CCS who typically dedicates approximately 30 minutes per case on average. In response to these challenges, investigators [9-11] have applied both rule-based algorithms and machine learning methods, such as recurrent neural network, long short-term memory, and bidirectional encoder representations from transformers (BERT), to classify patients with specific conditions. However, the performance of these approaches remains limited.

By contrast, to control the rising health care costs, health authorities in many countries have implemented the diagnosis related group (DRG) payment system. The NHIA in Taiwan has implemented the Taiwan DRG (Tw-DRG) payment systems since 2010 [12] to consolidate related DRGs for the purpose of determining payment standards [13]. Tw-DRGs are classified by major diagnostic categories (MDCs) that depend on the assigned *ICD-10-CM* codes. Consequently, accurate *ICD-10-CM* coding is critical for the accurate generation of Tw-DRGs, as coding errors can lead to inappropriate treatment options, delayed reimbursement processes, and significant financial losses for hospitals [14,15]. Factors including incomplete information, incorrect data entry, and insufficient coder expertise can lead to inaccurate coding [16]. In addition, errors may also arise from incorrect human perception [17], the complex technical nature of the work [18], and human fatigue from heavy workloads [19]. While natural language processing (NLP)–driven autocoding systems have the potential to enhance the quality of the manual coding results and expedite code assignment, it is imperative to assess their accuracy to ensure cost savings for the hospitals [20]. In summary, precise coding according to the *ICD-10-CM* system is essential for accurately categorizing Tw-DRGs, as any fault in this process can lead to misclassification and subsequently impact health care reimbursements.

### Study Overview

In this study, we developed 2 deep learning–based models, the hierarchical attention network (HAN) and the GPT-2 (OpenAI), in the manner of multi-label supervised learning for *ICD-10-CM* coding. The former is a conventional classification model, and the latter is categorized as a generative model.

To facilitate the coding process in the real hospital environment, we established an NLP-driven *ICD-10-CM* autocoding system. This system includes a user-friendly visual interface to display the predicted coding results, CIs, and relevant medical record keywords. We integrated the system into the coding procedure protocolled at Kaohsiung Medical University Chung-Ho Memorial Hospital (KMUCHH) to assist the workflow of clinical coders to expedite the efficiency of disease coding. We then compared the consistency of the principal diagnosis and MDC coding between the autocoding system and the data curated by the CCS.

## Methods

### Data Set and Task Definition

#### Data Collection and Preparation

This study used a total of 136,841 unstructured discharge summaries of patients who were hospitalized, recorded in the KMUCHH from April 1, 2019, to December 31, 2020, as the primary data source for the development of the *ICD-10-CM* coding system. After deploying the developed system in the workflow of the KMUCHH CSS, we collected an additional data set containing 2632 discharge cases processed by the system from February 2023, to April 2023, to assess the performance of the *ICD-10-CM* coding system in the real hospital environment and the feasibility of assisting the process of Tw-DRGs. The original data set contains 15,756 discharge cases. After excluding 7541 (47.86%) non–Tw-DRGs cases and 5583 (35.43%) with incomplete electronic medical records (EMRs), a data set of 2632 (16.7%) discharge cases was used for our evaluation.

To maximize the amount of data available for the training phase of our system, we composed a test set by selecting the latest 1000 discharge summaries from the raw data sorted by time stamp. The remaining summaries were then allocated for use as the training set. During the training phase, we randomly sampled 5% (6842/136,841) of the discharge summaries to form the validation set consisting of 6842 summaries. As depicted in Table 1, a total of 129,000 (94.27%) of 136,841 discharge summaries were assigned to the training set (comprising a total of 567,957 *ICD-10-CM* codes, with 11,494 (2.02%) being unique), 6842 (5%) discharge summaries were assigned to the validation set (with a total of 36,205 codes, including 4038, 11.15% unique ones), and 1000 (0.73%) discharge summaries were included in the test set with a total of 10,412 codes and 1482 (14.23%) of them being unique.

**Table 1:** Prevalence of the 2014 International Classification of Diseases, Tenth Revision, Clinical Modification codes used in the compiled data set consisting of 136,841 discharge summaries.

Chapters	Blocks	Definitions	Codes in training set (n=567,957), n (%)	Codes in validation set (n=36,205), n (%)	Codes in test set (n=10,412), n (%)
I	A00-B99	Certain infectious and parasitic diseases	35,708 (6.29)	2233 (6.17)	341 (3.28)
II	C00-D48	Neoplasms	82,248 (14.48)	4838 (13.36)	827 (7.94)
III	D50-D89	Diseases of the blood and blood-forming organs and certain disorders involving the immune mechanism	17,347 (3.05)	1281 (3.54)	168 (1.61)
IV	E00-E90	Endocrine, nutritional, and metabolic diseases	63,545 (11.19)	4077 (11.26)	301 (2.89)
V	F00-F99	Mental and behavioral disorders	8835 (1.56)	565 (1.56)	289 (2.78)
VI	G00-G99	Diseases of the nervous system	13,236 (2.33)	835 (2.31)	395 (3.79)
VII	H00-H59	Diseases of the eye and adnexa	8520 (1.5)	421 (1.16)	661 (6.35)
VIII	H60-H95	Diseases of the ear and mastoid process	1304 (0.23)	120 (0.33)	193 (1.85)
IX	I00-I99	Diseases of the circulatory system	82,005 (14.44)	5066 (13.99)	636 (6.11)
X	J00-J99	Diseases of the respiratory system	31,233 (5.5)	2091 (5.78)	285 (2.74)
XI	K00-K93	Diseases of the digestive system	48,827 (8.6)	3098 (8.56)	514 (4.94)
XII	L00-L99	Diseases of the skin and subcutaneous tissue	7469 (1.32)	488 (1.35)	372 (3.57)
XIII	M00-M99	Diseases of the musculoskeletal system and connective tissue	20,312 (3.58)	1296 (3.58)	1191 (11.44)
XIV	N00-N99	Diseases of the genitourinary system	39,504 (6.96)	2498 (6.9)	328 (3.15)
XV	O00-O99	Pregnancy, childbirth, and the puerperium	3887 (0.68)	255 (0.70)	394 (3.78)
XVI	P00-P96	Certain conditions originating in the perinatal period	4297 (0.76)	271 (0.75)	202 (1.94)
XVII	Q00-Q99	Congenital malformations, deformations, and chromosomal abnormalities	2918 (0.51)	171 (0.47)	315 (3.03)
XVIII	R00-R99	Symptoms, signs, and abnormal clinical and laboratory findings, not elsewhere classified	24,544 (4.32)	1636 (4.52)	392 (3.76)
XIX	S00-T98	Injury, poisoning, and certain other consequences of external causes	20,850 (3.67)	1783 (4.92)	1661 (15.95)
XX	V01-Y98	External causes of morbidity and mortality	11,791 (2.08)	711 (1.96)	530 (5.09)
XXI	Z00-Z99	Factors influencing health status and contact with health services	39,577 (6.97)	2471 (6.83)	417 (4)

#### ICD-10-CM Coding Task Formulation

The *ICD-10-CM* coding task presented in this study aims to develop an NLP autocoding system for generating *ICD-10-CM* codes from a patient’s discharge summary. An *ICD-10-CM* code consists of 3 to 7 characters, and each code begins with an alphabetic character that signifies the relevant classification chapter. The first 3 characters in the code designate the category of the diagnosis, while the subsequent 3 characters correspond to the related etiology. The seventh character provides the related extensions. As shown in Table 1, there are 21 chapters in *ICD-10-CM*. The ground-truth *ICD-10-CM* codes for the compiled 13,6841 discharge summaries were annotated by specialized CCSs at KMUCHH, culminating in a grand total of 11,653 unique codes. Textbox 1 displays an example of the discharge summary from the KMUCHH. The output of the coding system for each summary encompasses a main code along with multiple other codes, thereby formulating the task as a multi-labeling classification problem. Within our data set, the unique count for main codes and other codes is 5835 and 10,393, respectively. Moreover, the generated main codes will serve as the principal diagnosis, while the other codes will be used as secondary diagnoses in the Tw-DRGs estimation process.

**An example of the discharge summary from Kaohsiung Medical University Chung-Ho Memorial Hospital.**

Chief complaint:

Acute urine retention after discharge today

Impression on Admission:

Acute urine retention.

Bladder stone s/p op.

Hypertension.

Discharge Diagnosis:

--underlying--

#Acute urine retention.

#Hypertension.

#Hyperlipidemia

History on Admission:

This 71 y/o male has history of hypertension and under regular medical control. He was just discharged from our ward bladder stone and accepted surgical intervention (Endoscopic cystolithotripsy) in last admission. This time, he ...

### Data Preprocessing

During the preprocessing stage, delimiters (eg, “--underlying--” shown in Textbox 1) and prefix symbols (such as “#”) in the unstructured discharge summaries were filtered out. Subsequently, the clinical NLP toolkit was leveraged to preprocess the summaries by applying sentence splitting and tokenization [21].

### Deep Learning Models

As an effective approach to the coding task, we formulated it as a multi-label supervised learning problem and applied 2 deep learning–based methods to the compiled data set. The first model is grounded in HAN, a neural network architecture specifically crafted to tackle the complexities of modeling hierarchical structures within text data [22]. HAN leverages attention mechanisms to capture fine-grained information at both the document and sentence levels. The second model is a generative network built upon GPT-2; a causal language model released by OpenAI which was pretrained on large-scale text data [23]. The architecture is also featured with an attention mechanism that enables it to comprehend and generate natural language. HAN was selected for its capacity to provide high readability and interpretability of text through visualization [24]. It leverages hierarchical text representation and attention mechanisms to effectively highlight important words and sentences at multiple levels of the text. This enhances the interpretability of the model to allow a better understanding of its internal workings and gain insights into the model's decision-making process with a strengthened trust in its outputs [25]. Furthermore, considering the growing significance of generative artificial intelligence (AI) in research and landing applications, large language models such as GPT-2 are poised to bring about significant transformation in clinical medicine and health care and will be ubiquitous in these fields. However, concerns regarding data sensitivity, inference speed, hardware requirements, as well as the ease of deployment and difficulty of maintenance of the system are paramount [26]. In light of these considerations, we opted for the GPT-2 model due to its balance of performance and practicality in our real clinical environment setting.

**Figure 1:**
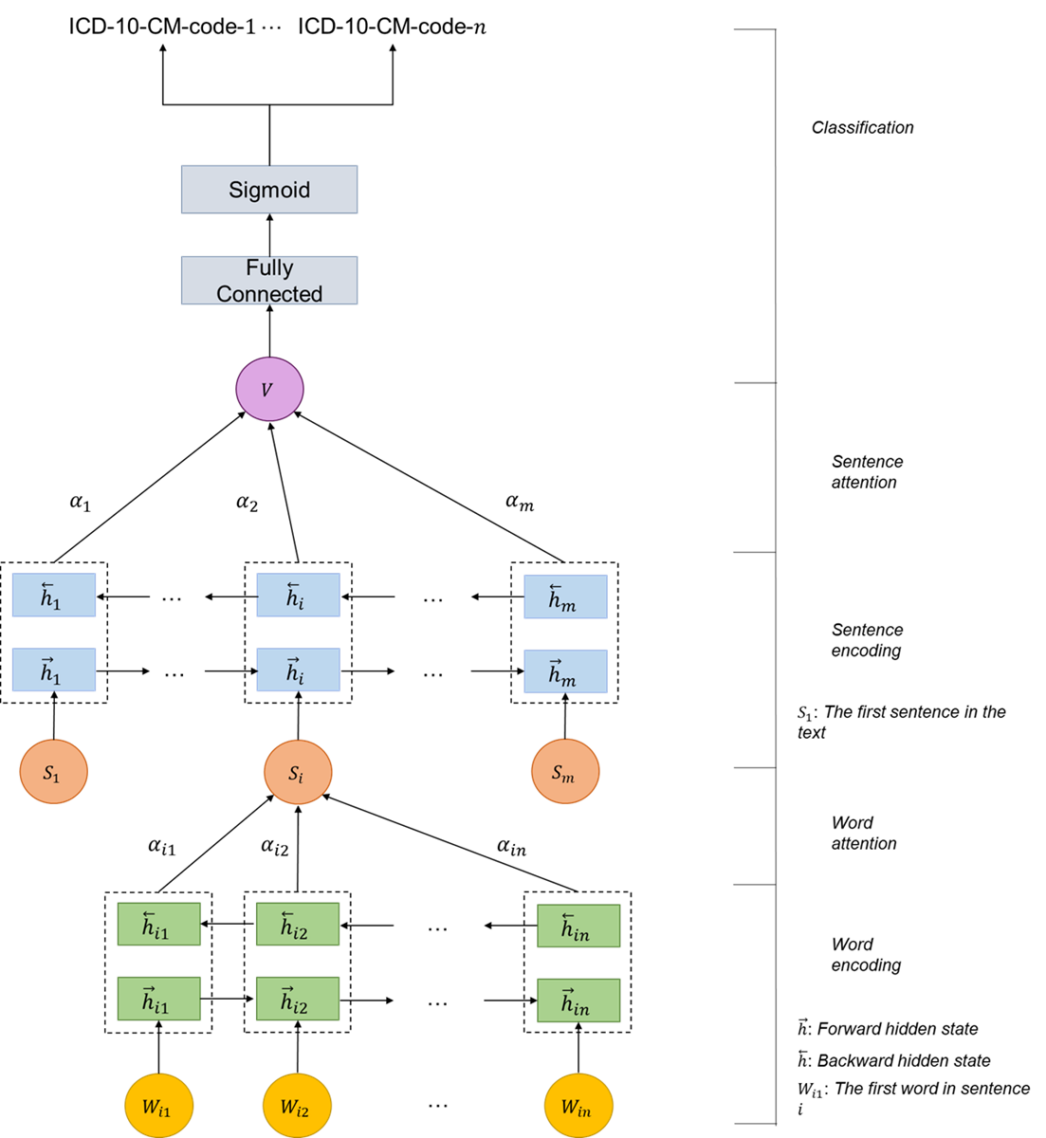
Architecture of the hierarchical attention network implementation in this study. ICD-10-CM: International Classification of Diseases, 10th Revision, Clinical Modification.



Our HAN implementation adheres to the original network architecture and is tailored specifically for the multi-labeling classification task of *ICD-10-CM* codes. The customization involves the application of a fully connected layer to transform the attention results of HAN into the desired number of target ICD codes as illustrated in Figure 1. The process begins in the embedding layer, where we use pretrained global vector embeddings containing 300D vectors trained on a corpus of 6 billion tokens [27] to extract essential information from the textual data. Following the embedding layer, we execute word-level and sentence-level encoding procedures, which use an attention mechanism to capture crucial words and sentences within the text. Subsequently, we implement a fully connected layer to generate a set of 11,653 unique *ICD-10-CM* codes. Finally, the output values are transformed into a range between 0 and 1 using a sigmoid layer, which represents the probability of the ICD code being related to the summary. The loss function used for the HAN model is the cross-entropy loss defined in Equation (1), which measures the sum of the negative log-likelihood of the probabilities of the actual labels. A large deviation between and the actual label cause a greater value in the loss and thus is penalized more during training.

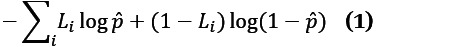



For inference, a threshold is set to assign the main code and other codes to the document based on the estimated label probability set following equations 2 and 3.

argmax(p) **(2)**

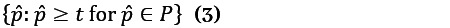



As shown in the aforementioned equations, the threshold *t* is applied to determine the predicted probability 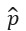 for *ICD-10-CM* codes, with the code having the highest probability selected as the *ICD-10-CM* main code. The main code is subsequently used as the principal diagnosis in the Tw-DRG process. After conducting multiple experiments, we set the threshold *t* in equation (3) as 0.5 as it has been determined that this setting achieves a better balance between the precision and recall.

**Figure 2:**
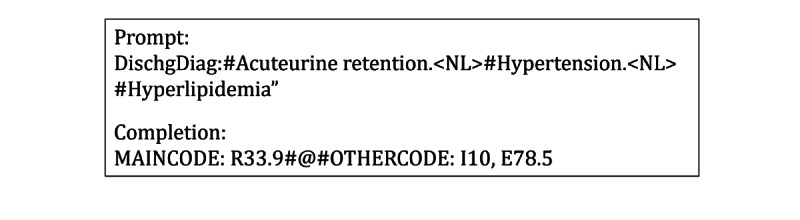
An example of fine-tuned data for GPT-2.



In our GPT-2 implementation, we used a pretrained model developed by Papanikolaou and Pierleoni [28], which was a model based on the GPT-2 architecture fine-tuned using 0.5 million PubMed abstracts with 355 million parameters. This model was adopted instead of the original GPT-2 released by OpenAI to address the issue of the variety of medical vocabularies conveyed in the summaries, which include diverse clinical data such as symptoms, diseases, and medications written in varying styles. To further fine-tune the pretrained model on our data set, we added special tokens to the model’s tokenizer to help the model understand the structure of the sequence to complete the coding tasks. A total of 3 special tokens including “CLS,” “SEP,” and “#@#” were defined, which represent the starting position symbol of the input, the ending position symbol of the sentence, and the separator between *ICD-10-CM* main code (MAINCODE) and other codes (OTHERCODE), respectively. An example of a fine-tuned instance after processing is shown in Figure 2. The coding task is then formulated in a generative manner that is given an input text (*eg*, the prompt part shown in Figure 2) with a sequence of n tokens = [x_1_,x_2_,...,x_n_], and the target output sequence y = [y_1_,y_2_,...,y_m_]. The objective is to maximize the conditional probability in the auto-regressive formulation represented in equation (4).

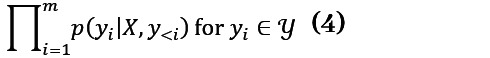



During inference, the probability of each generated code is estimated by averaging the accumulated conditional probabilities for the tokens of the generated code. In instances where discharge summaries exceed the 1024 token limit of the GPT-2 model, we truncate the text during preprocessing to ensure it stays within the specified limit.

Finally, in addition to HAN and GPT-2, we implemented 2 baselines for performance comparison, one is a BERT-based model proposed by Devlin et al [29] and the other is based on the bidirectional gated recurrent unit along with the BERT-based word representation architecture proposed by Chen et al [30].

### Integration of the Developed NLP-Driven AI-Assisted Coding System at KMUCHH

**Figure 3:**
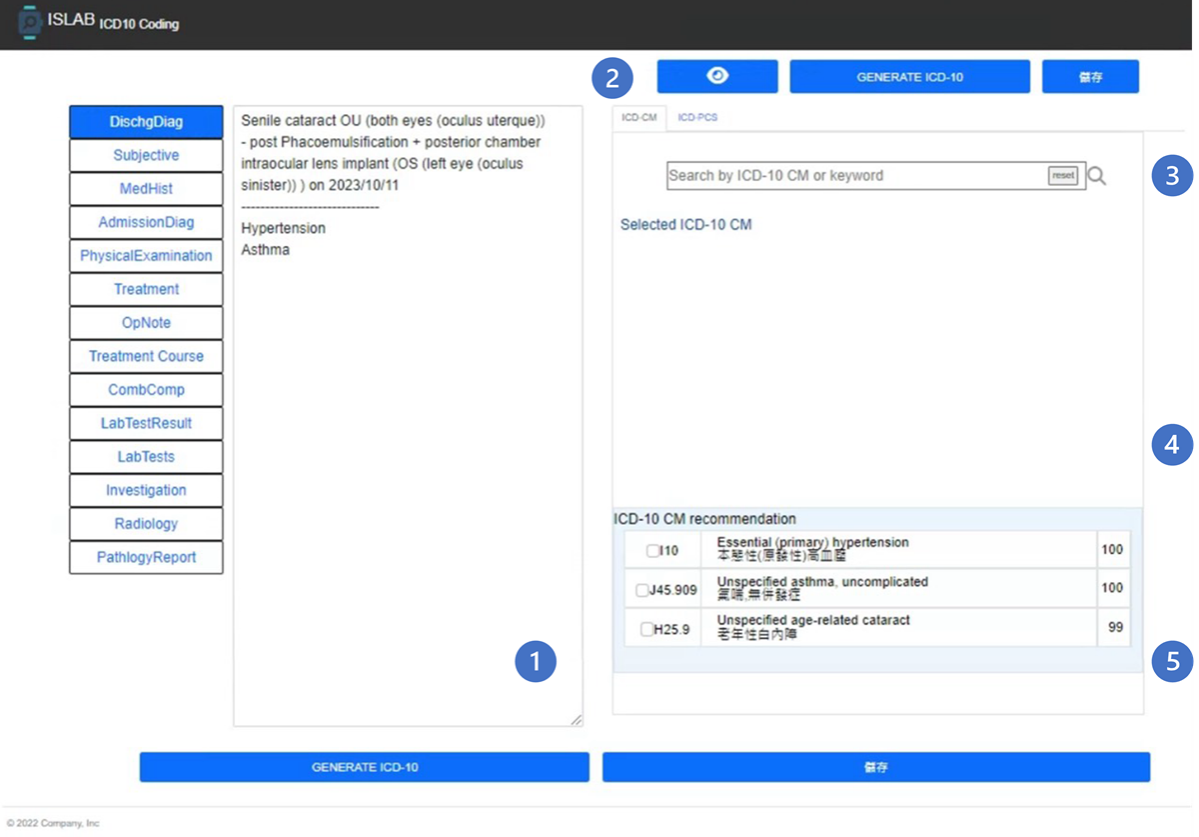
User interface of a natural language processing–driven artificial intelligence–assisted coding system at Kaohsiung Medical University Chung-Ho Memorial Hospital.

**Figure 4:**
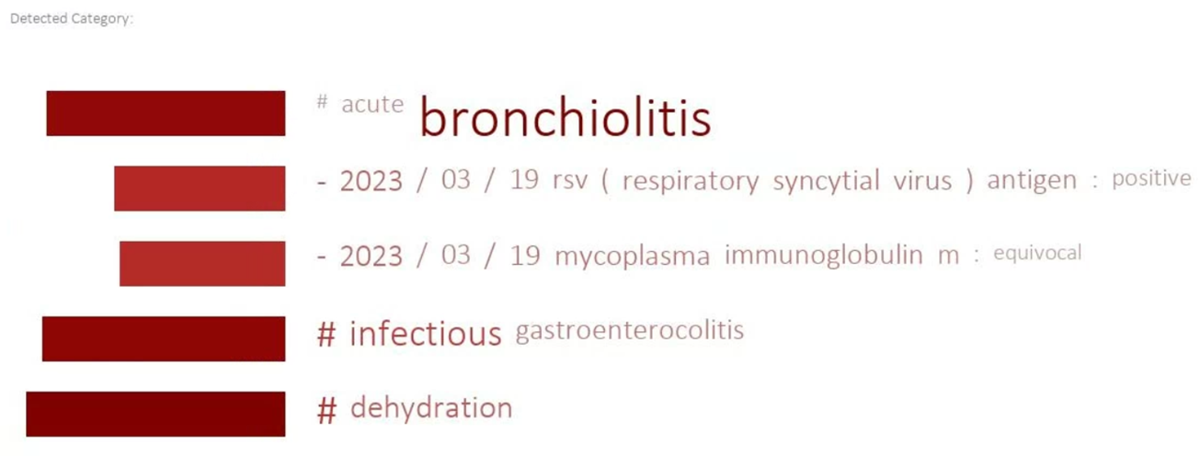
Visualization of the attentive key terms highlighted in a given discharge summary by the attention mechanism.

To integrate the developed models within the workflow of the CSS at KMUCHH, a user interface as illustrated in Figure 3 was implemented [31]. Various components can be identified within this interface. Area (1) serves as the patient EMRs display area. It conveniently showcases the relevant unstructured text content from the EMRs with 14 selectable sections. Area (2) features 3 functional buttons from left to right. The first button is used for attention visualization as presented in Figure 4. To display this figure, we analyze the results of the attention layer of the deployed model to highlight key medical terms from discharge summaries.

The second button initiates the *ICD-10-CM* automatic coding, which exploits the developed model to generate *ICD-10-CM* codes based on the content shown in area (1). The third button is the save button, which is used to store the results of *ICD-10-CM* autocoding, user selections, and system operation time stamps in the database. The search bar is located in area (3) and can be used by the CCS to manually look up *ICD-10-CM* codes or keywords in English to assist in manually adding missing codes. Any newly added codes are combined with the selected codes and displayed in area (4). Finally, area (5) provides a list of *ICD-10-CM* codes recommended by the deployed model through the coding assistant process. These results are presented in a checklist format as illustrated in Figure 3, including *ICD-10-CM* codes, their corresponding Chinese and English descriptions, and the confidence assigned by the deep learning model.

**Figure 5:**
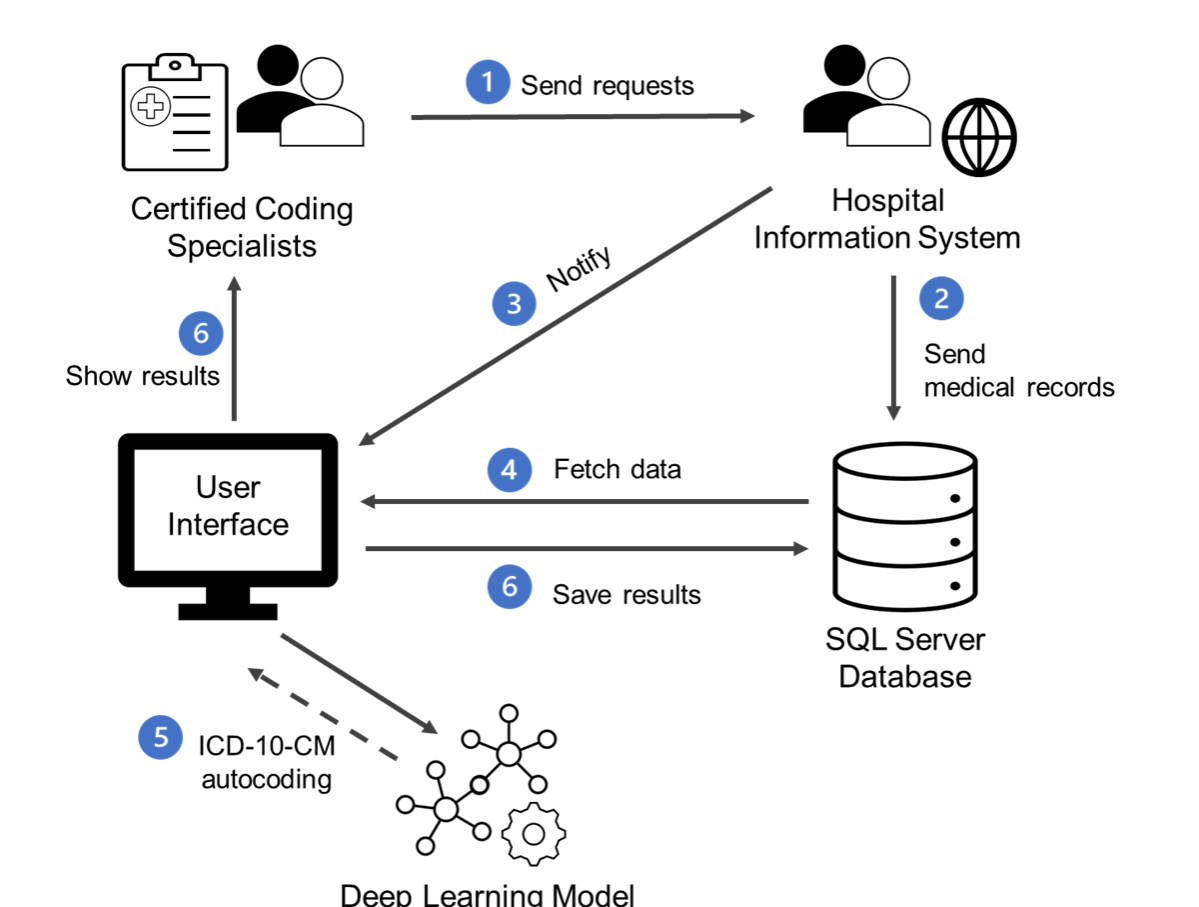
Workflow of the natural language processing–driven artificial intelligence–assisted coding system deployed in Kaohsiung Medical University Chung-Ho Memorial Hospital. ICD-10-CM: International Classification of Diseases, 10th Revision, Clinical Modification; SQL: structured query language.

The user interface and coding system were deployed and tested in January 2023 at KMUCHH. An overview of the integrated workflow is displayed in Figure 5. First, the CCS uses the user interface developed for the AI-assisted coding system to send requests to the hospital information system. Once the hospital information system accepts the request, the requested EMRs are transferred to the structured query language server database specifically developed for the study. Subsequently, a notification is dispatched to the user interface. Upon receiving the notification, the user interface retrieves the corresponding medical record from the database and uses the developed model to generate suggested *ICD-10-CM* codes. The recommendations are then presented in area (5) of the user interface as shown in Figure 3. Finally, the CCS reviews the recommendations and selects the final *ICD-10-CM* codes in area (4) of Figure 3, which are subsequently saved in the database.

### Tw-DRG Payment System

Figure S1 in Multimedia Appendix 1 depicts the current implementation practice of the Tw-DRG payment system. The initial step of this process involves the conversion of diagnoses from physician-provided discharge summaries into *ICD-10-CM* codes and follows the principal diagnosis selection principle defined by NHIA to use the main cause of hospitalization as the principal diagnosis. In cases where multiple principal diagnoses are identified simultaneously, the one with the highest medical cost will be selected. Our NLP-driven AI-assisted coding system plays a pivotal role in the first and second steps of the process, automating the *ICD-10-CM* coding process based on discharge summaries and determining the principal diagnosis.

As listed in Table 2, the cause of a patient’s hospitalization identified by our system as the principal diagnosis is subsequently categorized into the corresponding MDC under the Tw-DRG calculation software program. The MDC schematic classification for inpatient cases consists of 26 distinct categories, spanning from pre-MDC to MDC 1 through MDC 25. Once the principal diagnosis is determined for a patient, similar therapeutic diseases or procedures are further divided into multiple DRGs. This division considers various factors including the patient’s age and gender, the presence of comorbidities or complications (secondary diagnoses), discharge status, etc. The prospective inpatient costs that the NHIA should reimburse hospitals are calculated by leveraging historical data from the health care industry as a foundational reference. In sum, the DRG provides essential information for this specific hospitalization, including details related to health insurance reimbursement, relative weight, and the presence of comorbidities and complications.

**Table 2:** The 26 major diagnostic categories (MDC) in Taiwan diagnosis related groups.

MDC	Title
Pre	Heart Transplant Liver Transplant Bone Marrow Transplant Tracheostomy Lung Transplant Pancreatic Transplant
1	Diseases and Disorders of the Nervous System
2	Diseases and Disorders of the Eye
3	Diseases and Disorders of the Ear, Nose, Mouth and Throat
4	Diseases and Disorders of the Respiratory System
5	Diseases and Disorders of the Circulatory System
6	Diseases and Disorders of the Digestive System
7	Diseases and Disorders of the Hepatobiliary System and Pancreas
8	Diseases and Disorders of the Musculoskeletal System and Connective Tissue
9	Diseases and Disorders of the Skin, Subcutaneous Tissue and Breast
10	Endocrine, Nutritional and Metabolic Diseases and Disorders
11	Diseases and Disorders of the Kidney and Urinary Tract
12	Diseases and Disorders of the Male Reproductive System
13	Diseases and Disorders of the Female Reproductive System
14	Pregnancy, Childbirth and the Puerperium
15	Newborns and Other Neonates with Conditions Originating in the Perinatal Period
16	Diseases and Disorders of the Blood and Blood Forming Organs and Immunological Disorders
17	Myeloproliferative Diseases and Disorders, and Poorly Differentiated Neoplasm
18	Infectious and Parasitic Diseases (Systemic or Unspecified Sites)
19	Mental Diseases and Disorders
20	Alcohol/Drug Use and Alcohol/Drug Induced Organic Mental Disorders
21	Injuries, Poisonings and Toxic Effects of Drugs
22	Burns
23	Factors Influencing Health Status and Other Contacts with Health Services
24	Multiple Significant Trauma
25	Human Immunodeficiency Virus Infection

### Experiment Configurations

Both deep learning models were implemented using CUDA 12.0 (Nvidia Corp) and the PyTorch (Meta Platforms) libraries trained on machines equipped with an Intel i7-13700 processor (Intel Corporation), 64 GB of RAM, and an NVIDIA GeForce RTX 4090 24-GB graphics card (Nvidia Corp). During the training of these 2 models, different hyper-parameter configurations were used. For HAN, we set the batch size to 32, the learning rate to 1e-3, and the number of epochs to 500, and used the Adam optimizer [32] for optimization. During the training process, we implemented an early stopping strategy with a patience value of 50, which was triggered if there was no improvement in the *F*_1_-score or loss, or if the loss on the validation set reached 0. For GPT-2, we configured the learning rate to be 1.5e-4, set the number of epochs to 10, and set the batch size to 4 to prevent out-of-memory issues. We used the AdamW optimizer [33] for parameter optimization, setting the epsilon to 1.0e-09 to ensure that the model stops updating when the learning rate drops below this threshold. In addition, to align with the maximum input length in the GPT-2 architecture, we set the maximum input length for each training instance to 1024.

The evaluation metric used to assess the performance of the developed models is the *F*-measure, calculated from the harmonic mean of the precision and recall. Precision and recall are computed based on the counts of correctly predicted *ICD-10-CM* codes (true positives), incorrectly predicted *ICD-10-CM* codes (false positives), and undetected *ICD-10-CM* codes (false negatives). These values are determined by comparing the model’s predictions with the ICD codes assigned by the CCS. These metrics are calculated using the following equations 5, 6 and 7 for the precision, recall and *F*_1_-scores.



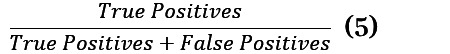





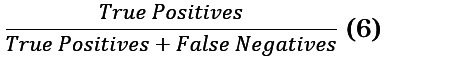





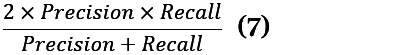



It is worth noting that in the evaluation step, in addition to assessing the performance of the autocoding systems on the original compiled corpus, we also compiled a new Tw-DRGs data set consisting of EMRs collected from the real KMUCHH environment. The new data set was processed by the aforementioned autocoding systems in series with NHIA’s DRG calculation software. In addition, to assess the reliability of our NLP-driven AI-assisted coding system in the Tw-DRG inpatient payment process, we used the κ measure as an indicator of agreement for MDC between our system and CCS curated results. Specifically, we used the Cohen κ [34] to estimate the κ values. For this comparison, we used the Tw-DRG calculation program provided by the NHIA in Taiwan. This program uses inputs, such as the predicted main code, other codes, gender, and date of birth to perform MDC classification and DRG grouping. We developed a program to analyze the output from the Tw-DRG calculation program for the comparison study.

### Ethical Considerations

This study was approved by the KMUCHH Institutional Review Board (protocol title: “Developing Artificial Intelligence model to support *ICD-10-CM* and PCS coding and comparing the performance between machine coding and manual coding”; institutional review board number: KMUHIRB-E(II)-20230214). As the study utilized secondary data collected under the original institutional review board approved protocol, no additional informed consent was required. All data collected from KMUCHH was aggregated for research purposes adhering to fair use principles. Participant privacy was rigorously protected through anonymization, and stringent confidentiality measures were implemented. Given that the study involved only the analysis of existing, deidentified data, there was no direct interaction with participants, and thus, no compensation was provided. Additionally, the study strictly adhered to all applicable local, national, regional, and international laws and regulations concerning the protection of personal information, privacy, and human rights.

## Results

### Overview

In the following subsections, we demonstrated the effectiveness of deploying NLP-driven AI-assisted coding systems, specifically using HAN and GPT-2 models, in the context of *ICD-10-CM* coding and Tw-DRGs process. We provide a comparative analysis of the AI-assisted coding results with CCS curated results for Tw-DRGs using the data set gathered from the deployment of the developed AI-assistant system in the hospital environment. This analysis validates the potential of NLP-driven AI-assisted coding in facilitating the Tw-DRGs process, highlighting its effectiveness in improving coding.

### Performance Comparison of the Developed Deep Learning Models on the ICD-10-CM Coding Task

Tables 3 and 4 offer a comprehensive analysis of the performance of the baseline models as well as the HAN and GPT-2 models in *ICD-10-CM* autocoding on the test set. In addition to the full code results, Table 3 shows the performance of each model on the top-50 codes (Top-50 F) which is often reported in previous research papers. The list of the top-50 *ICD-10-CM* codes can be found in Multimedia Appendix 2. The results presented in Table 3 highlight the superior performance of the GPT-2 model compared to the other models. GPT-2 exhibited higher precision, recall, and *F*-measure in its overall performance compared to HAN, with improvements of 0.134, 0.077, and 0.107, respectively. Additionally, the boost in the *F*_1_-score for the main code further emphasizes the advantage of applying the GPT-2 model over the other models. Both HAN and GPT-2 models achieved satisfactory top-50 *F*_1_-scores but obtained significantly lower *F*_1_-scores for the full codes owing to the long-tailed distribution of the *ICD-10-CM* codes assigned by the CCS.

**Table 3:** Overall performance evaluation of hierarchical attention network (HAN) and GPT-2 models in the International Classification of Diseases, 10th Revision, Clinical Modification, autocoding on the test set.

Model	Precision	Recall	*F*-measure	Top-50 (F)	Main code (F)
HAN	0.529	0.594	0.560	0.818	0.429
GPT-2	0.663	*0.671* ^*a*^	*0.667* ^*a*^	*0.851* ^*a*^	*0.575* ^*a*^
BiGRU^b^	*0.765* ^*a*^	0.451	0.567	0.717	0.250
BERT^c^	0.759	0.366	0.494	0.698	0.215

^a^The best value for each performance metric is italicized.

^b^BiGRU: bidirectional gated recurrent unit.

^c^BERT: bidirectional encoder representations from transformers.

Table 4 provides a more detailed breakdown of the *ICD-10-CM* subcategory performance for the 2 models. The results underscore the consistent superiority of GPT-2 over HAN across all categories, particularly in situations with limited data (<10%). Notably, GPT-2 acquired better *F*_1_-scores in the T, H, and O categories compared to HAN with an increase of 0.387, 0.279, and 0.269, respectively. This accentuates the advantage of GPT-2 due to its pretrained nature, which enables it to extract crucial features in a more efficient manner compared to HAN when only limited training data are provided.

By contrast, both models revealed low performance in the X and Y categories. These 2 categories suffered from a lack of data, as they have the lowest number of training instances as outlined in the last column of Table 4. The restricted amount of training data poses a challenge to the models in achieving better performances in these specific categories.

**Table 4:** The International Classification of Diseases, Tenth Revision, Clinical Modification, category-specific performance comparison of the hierarchical attention network (HAN) and GPT-2 on the test set.

Categories (block)	Chapters	Models (*F*-measure)		Discharge summaries in the training set (n=105,101), n (%)
		HAN	GPT-2	
A	I	0.851	*0.955* ^a^	15,267 (14.53)
B	I	0.703	*0.889* ^a^	20,441 (19.45)
C	II	0.812	*0.890* ^a^	71,202 (67.75)
D	II, III	0.702	*0.814* ^a^	28,393 (27.01)
E	IV	0.797	*0.888* ^a^	63,545 (60.46)
F	V	0.611	*0.833* ^a^	8835 (8.41)
G	VI	0.566	*0.794* ^a^	13,236 (12.59)
H	VII, VIII	0.576	*0.855* ^a^	9824 (9.35)
I	IX	0.781	*0.833* ^a^	82,005 (78.02)
J	X	0.756	*0.880* ^a^	31,233 (29.72)
K	XI	0.714	*0.843* ^a^	48,827 (46.46)
L	XII	0.496	*0.730* ^a^	7369 (7.01)
M	XIII	0.549	*0.764* ^a^	20,312 (19.33)
N	XIV	0.783	*0.894* ^a^	39,504 (37.59)
O	XV	0.583	*0.852* ^a^	3887 (3.7)
P	XVI	0.738	*0.857* ^a^	4297 (4.09)
Q	XVII	0.571	*0.652* ^a^	2918 (2.78)
R	XVIII	0.545	*0.617* ^a^	24,544 (23.35)
S	XIX	0.661	*0.742* ^a^	20,850 (19.84)
T	XIX	0.297	*0.684* ^a^	8505 (8.09)
V	XX	0.446	*0.584* ^a^	3613 (3.44)
W	XX	0.459	*0.619* ^a^	3558 (3.39)
X	XX	0.270	*0.444* ^a^	1968 (1.87)
Y	XX	0.425	*0.458* ^a^	2652 (2.52)
Z	XXI	0.657	*0.747* ^a^	39,577 (37.66)

^a^The best value for each performance metric is italicized.

### Comparative Analysis of NLP-Driven AI-Assisted Coding Results With CCS for Tw-DRGs in the Real Hospital Environment

In this subsection, we provide a comparative analysis of the performance of the deployed systems from the perspective of Tw-DRGs. The performance of Tw-DRGs was estimated on the additional data set of 2632 discharge cases stored in the SQL server database indicated in Figure 5, which was collected from February 2023 to April 2023.

As described in the previous section on the Tw-DRGs payment system, the principal diagnosis is the crucial factor in determining the MDC, while secondary diagnoses are only used to determine different Tw-DRGs distributions within the same MDC. The principal diagnosis is determined based on the reason for the patient’s hospitalization and only a single disease can be selected. If there are multiple diseases for which the patient is receiving treatment upon admission, selecting any one of them as the principal diagnosis is not considered an error but may affect the results of Tw-DRGs. Hence, the principal diagnosis selection cannot be assessed by solely comparing the system output against the *ICD-10-CM* main codes initially assigned by the CCS. Instead, it necessitates the expertise of the CCS to reevaluate both the EMRs and the codes proposed by the developed systems. Therefore, to provide a comparative analysis of the 2 developed models in terms of their performance in defining the principal diagnosis as shown in Table 5, the senior CCS, author ATL, who is the third author of the paper, examined the discharged cases to determine the accuracy of principal diagnoses suggested by the AI-assisted coding systems based on the HAN and GPT-2 models.

**Table 5:** Comparison of performance between the deep learning models and certified coding specialist–curated results for principal diagnosis in discharge cases.

Models and conditions			February 2023 (n=748), n (%)		March 2023 (n=991), n (%)		April 2023 (n=893), n (%)
**HAN^a^ (*F*-measure=0.524)**							
	All correct^b^	79 (10.6)		181 (18.3)		177 (19.8)	
	Principal diagnosis with incorrect secondary diagnoses^c^	462 (61.8)		477 (48.1)		369 (41.3)	
	No principal diagnosis^d^	181 (24.2)		277 (27)		285 (31.9)	
	All incorrect^e^	26 (3.5)		56 (5.5)		62 (6.9)	
**GPT-2 (*F*-measure=0.621)**							
	All correct^b^	130 (17.4)		161 (16.2)		144 (16.1)	
	Principal diagnosis with incorrect secondary diagnoses^c^	443 (59.2)		599 (60.4)		524 (58.7)	
	No principal diagnosis^d^	165 (22.1)		186 (18.8)		183 (20.5)	
	All incorrect^e^	24 (3.2)		29 (2.9)		43 (4.8)	

^a^HAN: hierarchical attention network.

^b^Autocoding results were identical to certified coding specialists.

^c^Autocoding system correctly identified the principal diagnosis, but discrepancies exist in ≥1 secondary diagnosis codes assigned by certified coding specialists.

^d^Autocoding system’s principal diagnosis was different from certified coding specialists.

^e^Autocoding results were entirely different from certified coding specialists.

The first column of Table 5 shows the *F*-measure estimates for HAN (0.524) and GPT-2 (0.621) in their suggestion for main codes in comparison with the CCS coding results. The reported *F*-measures align with our observation on the test set and validate GPT-2 as a more reliable model. The manual reassessment of the results performed by the senior CCS according to the definition of principal diagnosis is displayed in the second to fourth columns of the table. Notably, most (1308/2632, 49.7%) of the cases of the 2 deep learning models when reviewed against the manual coding of the CCS fall under the category “principal diagnosis with incorrect secondary diagnoses,” followed by the category “no principal diagnosis.” The “all incorrect” cases had the lowest proportion among all categories. Predictions of GPT-2 achieved a better “all correct” category coverage and once again demonstrate superiority over HAN in facilitating the ICD coding task. The respective overall correct rate for principal diagnosis for HAN and GPT-2, which takes into account both categories “all correct” and “principal diagnosis with incorrect secondary diagnoses,” was 0.663 and 0.7602.

In Table 6, we extended the comparison to assess the agreement of MDC between the 2 developed models and the CCS. The first, second, and third columns of Table 6 show the MDC estimated results for the codes assigned by CCS, HAN, and GPT-2. The last 2 columns show the estimated κ values corresponding to the 2 models against the CCS.

**Table 6:** Comparison of the κ values between 2 deep learning models and a senior certified coding specialist (CCS) for major diagnostic category (MDC) in discharge cases spanning from February 2023 to April 2023 at Kaohsiung Medical University Chung-Ho Memorial Hospital.

MDC	CCS	Model			κ value^a^	
	Manual coding (n=2632), n (%)	HAN^b^ (n=2632), n (%)	GPT-2 (n=2632), n (%)	HAN (average 0.592)		GPT-2 (average 0.714)
Pre	0 (0)	0 (0)	0 (0)	—^c^		—
1	509 (19.34)	280 (10.64)	497 (18.88)	0.670^d^		0.803^d^
2	38 (1.44)	9 (0.34)	28 (1.06)	0.300		0.724^d^
3	132 (5.02)	113 (4.29)	124 (4.71)	0.689^d^		*0.852* ^e^
4	302 (11.47)	309 (11.74)	287 (10.90)	*0.845* ^e^		*0.826* ^e^
5	184 (6.99)	310 (11.78)	213 (8.09)	0.607		0.786^d^
6	229 (8.7)	217 (8.24)	222 (8.43)	0.775^d^		*0.818* ^e^
7	84 (3.19)	90 (3.42)	77 (2.93)	0.710^d^		*0.827* ^e^
8	87 (3.31)	66 (2.51)	87 (3.31)	0.576		0.786^d^
9	116 (4.41)	83 (3.15)	95 (3.61)	0.692^d^		0.729^d^
10	132 (5.02)	237 (9)	167 (6.34)	0.505		0.642^d^
11	120 (4.56)	205 (7.79)	99 (3.76)	0.648^d^		0.738^d^
12	3 (0.11)	7 (0.27)	3 (0.11)	0.362		0.666^d^
13	8 (0.30)	8 (0.30)	10 (0.38)	0.749^d^		*0.889* ^e^
14	41 (1.56)	11 (0.42)	38 (1.44)	0.419		*1.000* ^e^
15	15 (0.57)	9 (0.34)	16 (0.61)	0.635^d^		0.708^d^
16	57 (2.17)	64 (2.43)	44 (1.67)	0.624^d^		0.728^d^
17	2 (0.08)	3 (0.11)	1 (0.04)	0.399		0.666^d^
18	505 (19.19)	465 (17.67)	483 (18.35)	*0.870* ^e^		0.777^d^
19	0 (0)	23 (0.87)	3 (0.11)	—		—
20	0 (0)	2 (0.08)	2 (0.08)	—		—
21	21 (0.8)	24 (0.91)	16 (0.61)	0.404		0.646^d^
22	1 (0.04)	0 (0)	0 (0)	—		—
23	42 (1.6)	24 (0.91)	43 (1.63)	0.366		0.398
24	4 (0.15)	6 (0.23)	3 (0.11)	0.599		–0.010
25	0 (0)	2 (0.08)	0 (0)	—		—
None	0 (0)	65 (2.47)	74 (2.81)	—		—

^a^The average κ values are calculated using arithmetic averaging of the MDC categories, excluding the blank cells.

^b^HAN: hierarchical attention network.

^c^Not applicable.

^d^The κ value falls within the range of 0.61 to 0.80, indicating substantial reliability.

^e^The κ value falls within the range of 0.81 to 1.00, indicating almost perfect reliability.

Our analysis revealed that the CCS did not assign any cases to pre-MDC, MDC 19, MDC 20, and MDC 25 for the curated patients. By contrast, HAN erroneously assigned 23 (0.87%) of the 2632 cases to MDC 19, 2 (0.08%) cases to MDC 20, and 2 (0.08%) cases to MDC 25. GPT-2 made fewer mistakes in classifying 3 (0.11%) of the 2632 cases to MDC 19 and 2 (0.08%) cases to MDC 20. Following an analysis conducted by using SPSS (version 19; IBM Corp), outputs of both models along with the coding results manually curated by the CCS underwent Cohen κ evaluation for agreement. The results indicated that the average κ value for GPT-2 (0.714/Substantial) was approximately 12.2 percentage points higher than that of HAN (0.592/Moderate). Notably, the average κ value of GPT-2 exceeded 0.81 (highlighted in italics in Table 6) across 6 categories of the MDC, indicating almost perfect agreement between the CCS and GPT-2. These observations exhibit the effectiveness of NLP-driven systems in supporting the work of CCSs. It is worth mentioning that the κ value of GPT-2 in MDC 24 (pertaining to injuries involving 2 or more body systems) was recorded as –0.01 (poor). A close examination of the 7 cases of the compiled data set in MDC 24 reveals that while 6 (86%) cases shared consistent principal diagnoses, discrepancies arose in the secondary diagnoses. These discrepancies involved instances of overcoding for lung contusion and traumatic hemothorax, as well as undercoding for hepatic contusion and cervical spinal cord injury. Consequently, these inconsistencies resulted in a complete mismatch between the MDC results of GPT-2 and CCS-curated results.

### The Effectiveness of Applying Models Acquired With Biomedical Knowledge

We conducted an ablation study comparing the performance of 2 fine-tuned GPT-2 models: one fine-tuned on PubMed documents and the other being the original GPT-2 model released by OpenAI. This study aimed to assess the necessity of using models fine-tuned on biomedical documents. The results indicate that the PubMed fine-tuned model achieves slightly better performance than the original GPT-2 model, with *F*_1_-score improvements of 0.05 for full coding and 0.03 for main coding.

In addition, we used the following prompt template to assess the knowledge of the 2 GPT-2 models for the tail-50 ICD codes.

“<*ICD-10-CM* Code> < *ICD-10-CM* Description> The condition involves...”

From the generated texts, we observed that both GPT-2 models generally possess a considerable understanding of *ICD-10-CM* terms. For instance, when prompted with the *ICD-10-CM* code “C9502: Acute leukemia of unspecified cell type, in relapse,” the GPT-2 model generated the following response: “This condition involves erythroid and myeloid cells and is associated with a poor prognosis.” This response accurately describes a challenging scenario in leukemia management, where the disease has relapsed with the involvement of both erythroid and myeloid cell lines, often indicating a poor prognosis.

## Discussion

### Error Analysis

Ji et al [35] discussed several challenges encountered during the implementation of automated clinical coding procedures. One significant challenge is that electronic health records often contain a variety of professional medical vocabularies alongside noisy information, including nonstandard synonyms and misspellings. To address this issue, our study used a variation of GPT-2 fine-tuned using 0.5 million PubMed abstracts to enhance its ability to recognize medical terminologies. The observations from the prompting results, as described in the Methods section, highlight the biomedical knowledge acquired by the pretrained GPT-2 model. This enhancement enabled the GPT-2 model perform better than the HAN model, which was not pretrained on biomedical data, particularly in categories, such as XIX-T, VII, VIII, and XV, where training instances were limited. Our analysis also reveals that HAN tends to attend to noisy information and sometimes generates completely irrelevant codes, which can be frustrating for CCSs. The result underscores the GPT-2 model’s adeptness in comprehending medical context and its effectiveness in mitigating the challenges associated with clinical coding procedures.

Another problem highlighted by Ji et al [35] is the high-dimensionality of medical codes and the long-tailed distribution, which results in limited corresponding training instances that are necessary for effective model training. EMRs associated with multiple diagnoses are particularly complex issues known as a multi-label extreme classification problem characterized by a vast label set, which was also encountered by our HAN model. In our implementation of HAN, the high-dimensional label space comprises 11,653 codes, magnifying the complexity of the task. On the contrary, while the high-dimensionality issue does not significantly impact the GPT-2 model, the scarcity of training instances still presents a considerable challenge. For instance, codes associated with the rarity of congenital conditions, such as those in category XVII (congenital malformations, deformations, and chromosomal abnormalities), are less frequent in the data set. This rarity results in fewer cases for the models to learn from, thereby hindering accurate predictions. In addition, we noted that certain codes manually labeled by CCSs, such as D489, K5100, Z8616, and others, were not present in the training set. Consequently, both models struggled to predict these unlearned codes, illustrating a critical limitation in the presented training data.

In addition, there are categories with abundant training instances where both models’ performance remains unsatisfactory. Notably, codes from category XVIII appear in 23.35% of the training set summaries, yet the *F*_1_-scores for both models are below 0.65. Upon analyzing the predicted results, we observed that while codes from the chapter, symptoms, signs, and abnormal clinical and laboratory findings, frequently appear in discharge summaries, disease classification rules often do not require separate coding for symptoms and signs that are related to a diagnosed disease. This discrepancy can lead to confusion for the developed models, causing them to misinterpret these entries.

To tackle this issue, we intend to augment the training set by incorporating additional coded content curated by the CCS in future iterations. This will enable our system to learn novel coding content that the system did not previously come across. Moreover, we plan to explore the use of ICD code representation methods, as proposed by Vu et al [36] and Wu et al [37], into our models. By integrating these methods, we aim to further enhance our system’s performance and robustness in addressing the challenges associated with automated clinical coding.

Moreover, although models with neural attentions learned to infer implicit relationships in discharge summaries by interpreting contextual expressions with weighted attentions, there are instances where pertinent information required for the coding judgment criteria is absent from the discharge summaries, for example, for chapters XIX and XX (injury, poisoning, and certain other consequences of external causes and external causes of morbidity and mortality). According to the classification rules, codes from these chapters are often used in conjunction with other codes. The data sources for these codes are not limited to discharge summaries but also include nursing records, imaging reports, and emergency department records. The developed models’ poor performance in these chapters may be due to the complexity and the need to integrate information from various sources, which is currently unavailable in the current implementation.

Finally, we noticed that certain codes only existed in the test set. These scenarios can lead to incorrect *ICD-10-CM* codes generated by the model. Some examples as such are listed subsequently.

In the discharge diagnosis, the developed system predicted the *ICD-10-CM* code for “# Heart failure” as I509:

# Dyspnea with desaturation, focus on HAP (hospital-acquired pneumonia) and coronavirus disease of 2019 # Sepsis, focus on HAP (hospital-acquired pneumonia) and catheter related UTI (urinary tract infection) # Hyponatremia # Heart failure.

However, the correct coding is I5020. This discrepancy is primarily due to supplementary information recorded in the medical history on admission, specifically mentioning “# chronic systolic heart failure.” Following discussions with the CCS, it was agreed that future enhancements will attempt to merge the content from the history on admission to enable the model to learn from a broader range of medical history information, thus ensuring more accurate coding outcomes.

### Comparison With Prior Work

Li et al [38] introduced the DeepLabeler, a deep learning architecture based on a combination of the convolutional neural network (CNN) with the document-to-vector technique [39] to extract and encode local and global features for *ICD-9* coding. Their approach achieved micro *F*-measures of 0.335 and 0.408 in the public multiparameter intelligent monitoring in intensive care (MIMIC)-II and MIMIC-III data sets, respectively. Zeng et al [40] transferred the knowledge learned from the Medical Subject Headings indexing domain using the large-scale biomedical semantic indexing competition challenge data set [41] to enhance the performance of the developed multi-scale CNN for automatic *ICD-9* coding. Their approach achieved a micro *F*-measure of 0.420 on the public MIMIC-III data set. Chen et al [30] used diagnostic records from the National Taiwan University Hospital to build a data set with a total of 1,043,124 labels (using 14,602 unique codes as prediction candidates) and developed a deep neural network classification model based on the bidirectional gated recurrent unit along with the BERT-based word representation method. This model obtained an *F*-measure of 0.715 for *ICD-10-CM* coding on their test set. Wu et al [37] proposed a pseudo label-wise attention mechanism aimed at automatically combining attention modes of similar ICD codes to tackle the issue of unbalanced *multi-label* classification in ICD coding. Their methodology involved using a bidirectional long short-term memory in tandem with the pseudo label-wise attention mechanism to represent EMRs as vectors. They then used a BERT-based pretrained model to determine the vector representations of the *ICD-10* codes. Finally, they calculated the similarity between EMR vectors and ICD vectors to determine the assigned codes. This approach yielded a micro *F*-measure of 0.806 on their private Chinese Xiangya data sets. In a separate study, Bhutto et al [42] proposed a deep recurrent-CNN architecture with a lambda-scaled attention module. Their approach yielded micro *F*-measures of 0.862 and 0.705 on a private Pakistan clinical notes. In comparison with these studies, the GPT-2 model in our research demonstrates competitive performance, particularly excelling in cases where the training data for specific categories is limited, showcasing its robustness in handling diverse and challenging scenarios.

In addition, certain previous research also explored the impact of erroneous *ICD-10-CM* coding on hospital finances. Zafirah et al [43] studied the potential loss attributable to clinical coding errors in a Malaysian teaching hospital. Their findings indicate a high prevalence of error coding in medical records, particularly concerning secondary diagnosis codes, which reached 81.3% (377/464). The estimated financial impact on the medical discipline in this hospital amounted to a potential profit loss of RM 85,804.92 (US $19,617.05). Toner et al [15] conducted a retrospective comparative analysis of case records for patients with the M966 diagnosis code (periprosthetic fracture) in a district general hospital. Their work revealed that *ICD-10-CM* coding errors resulted in a loss of £25,000 (US $33,029.97) when compared to the actual hospital revenue. In contrast with relevant studies, our examination of the consistency assessment of the autocoding system and CCS coding indicates promising prospects in reducing manual workload and providing coding references to minimize human errors. The error rate of CCS detected with the assistance of the AI-assisted coding system is 1.9% (50/2632; number of coding errors/total cases).

Pivotally, our work serves as the first endeavor to examine the feasibility of the combination of *ICD-10* coding with DRGs in the real hospital environment, which indicates a significant advancement in the area. In conclusion, the implementation of NLP-driven AI-assisted coding systems contributes to a reduction in CCS coding errors and manual workload, thereby enhancing the overall efficiency of the coding process, lowering error rates, and mitigating financial losses.

### Limitations

While our study demonstrates the potential of NLP-driven AI-assisted coding systems in improving *ICD-10-CM* coding accuracy and efficiency, several limitations should be acknowledged. First, the data set used for this study was sourced from a single hospital, which may limit the generalizability of the presented results. Future studies should include data from multiple hospitals, covering diverse geographical regions and varying patient demographics, to ensure broader applicability of the findings and to validate the robustness of the models across different settings.

Second, this study involved only 1 senior CCS to conduct the κ analysis for the agreements among the developed models. This limitation means that the results may not necessarily extend to other CCSs, particularly those with different levels of experience. Future works should consider recruiting a larger and more diverse group of CCSs, including both senior and junior coders, to evaluate the helpfulness of the proposed models comprehensively. In addition, studying the reduction in coding time and the impact on workflow efficiency in practical settings would provide valuable insights into the real-world benefits of AI-assisted coding systems.

Third, this study concentrated solely on Tw-DRGs, which are specific to Taiwan’s health care system. As a result, the findings may not be directly generalizable to other DRG systems used in different countries or regions, or to DRGs estimated in different periods. Further research is needed to ascertain the applicability of our conclusions to other DRG systems worldwide. Investigating the performance of the models in different international contexts and updating the models to reflect changes in DRG systems over time would enhance the relevance and utility of the findings.

### Conclusions

In summary, our study has demonstrated the effectiveness of deploying NLP-driven AI-assisted coding systems, specifically using HAN and GPT-2 models, in the context of *ICD-10-CM* coding and Tw-DRGs process. The comparative analysis revealed that GPT-2 consistently performed better than HAN and exhibited higher precision, recall, and *F*_1_-scores. This superior performance was particularly evident in scenarios with limited data, highlighting the robustness of GPT-2 in extracting vital features from discharge summaries. Moreover, the evaluation of principal diagnosis and MDC in Tw-DRGs showcased the utility of the developed models. GPT-2, in particular, acquired higher agreement values and made fewer mistakes in MDC classification. In the practical deployment of the system within a hospital environment, the comparative analysis with CCS validated the potential of NLP-driven autocoding in the Tw-DRGs process. Despite encountering certain discrepancies, our study demonstrates the significant value of the implemented models as tools that offer insights and support to CCSs during the coding process. The deployment of AI-assisted coding systems has the potential to enhance coding accuracy while simultaneously reducing manual workload, leading to improved process efficiency, lower error rates, and ultimately, a decrease in financial losses.

While our proposed system effectively alleviates the manual workload of CCSs, our error analysis has also unveiled notable challenges. These include the absence of coding judgment information in discharge summaries, presence of coding answers not included in the training set, and the need for suggesting 1 or more main codes for the development of *ICD-10* coding system to assist in the DRG process. These findings underscore areas for further improvement and refinement in future iterations of our system. Addressing these challenges will be pivotal in enhancing the efficacy and reliability of automated coding systems, thereby maximizing their potential to support and streamline both clinical coding and DRG processes.

## Appendix

Multimedia Appendix 1 Supplementary data for the process of Taiwan diagnosis related groups.

Multimedia Appendix 2 Supplementary data for the top 50 International Classification of Diseases, Tenth Revision, Clinical Modification, codes.

## Abbreviations

AI: artificial intelligence
BERT: bidirectional encoder representations from transformers
CCS: certified coding specialist
CNN: convolutional neural network
DRG: diagnosis related group
EMR: electronic medical record
HAN: hierarchical attention network
ICD: International Classification of Diseases
ICD-9: International Classification of Diseases, Ninth Revision
ICD-10-CM: International Classification of Diseases, Tenth Revision, Clinical Modification
KMUCHH: Kaohsiung Medical University Chung-Ho Memorial Hospital
MDC: major diagnostic category
MIMIC: multiparameter intelligent monitoring in intensive care
NHIA: National Health Insurance Administration
NLP: natural language processing
Tw-DRG: Taiwan Diagnosis Related group

## References

[1] (1978). International classification of diseases:[9th] ninth revision, basic tabulation list with alphabetic index. World Health Organization.

[2] Sivashankaran S, Borsi JP, Yoho A (2019). Have ICD-10 coding practices changed since 2015?. AMIA Annu Symp Proc.

[3] de Lissovoy G (2020). Codes, coding, and COVID-19. Med Care.

[4] Steindel SJ (2010). International classification of diseases, 10th edition, clinical modification and procedure coding system: descriptive overview of the next generation HIPAA code sets. J Am Med Inform Assoc.

[5] Mills RE, Butler RR, McCullough EC, Bao MZ, Averill RF (2011). Impact of the transition to ICD-10 on Medicare inpatient hospital payments. Medicare Medicaid Res Rev.

[6] Lee LM, Thacker SB (2011). Public health surveillance and knowing about health in the context of growing sources of health data. Am J Prev Med.

[7] Uzkuraitis C, Hastings K, Torney B (2010). Casemix funding optimisation: working together to make the most of every episode. Health Inf Manag.

[8] Ho C, Guilcher SJ, McKenzie N, Mouneimne M, Williams A, Voth J, Chen Y, Cronin S, Noonan VK, Jaglal SB (2017). Validation of algorithm to identify persons with non-traumatic spinal cord dysfunction in Canada using administrative health data. Top Spinal Cord Inj Rehabil.

[9] Shivade C, Raghavan P, Fosler-Lussier E, Embi PJ, Elhadad N, Johnson SB, Lai AM (2014). A review of approaches to identifying patient phenotype cohorts using electronic health records. J Am Med Inform Assoc.

[10] Liao KP, Cai T, Savova GK, Murphy SN, Karlson EW, Ananthakrishnan AN, Gainer VS, Shaw SY, Xia Z, Szolovits P, Churchill S, Kohane I (2015). Development of phenotype algorithms using electronic medical records and incorporating natural language processing. BMJ.

[11] Banda JM, Seneviratne M, Hernandez-Boussard T, Shah NH (2018). Advances in electronic phenotyping: from rule-based definitions to machine learning models. Annu Rev Biomed Data Sci.

[12] Wu JJ (2015). Implementation and outcome of Taiwan diagnosis-related group (DRG) payment system. Georgia State University.

[13] Wu CY, Chien LC, Lin CC, Ma HM, Hu RH, Chen CL, Lin T (2023). Original article: the impacts of DRG payment system on financial balance of multiple trauma: experiences of three trauma centers in Taiwan. Injury.

[14] Ayub S, Scali ST, Richter J, Huber TS, Beck AW, Fatima J, Berceli SA, Upchurch GR, Arnaoutakis D, Back MR, Giles KA (2019). Financial implications of coding inaccuracies in patients undergoing elective endovascular abdominal aortic aneurysm repair. J Vasc Surg.

[15] Toner E, Khaled A, Ramesh A, Qureshi MK, Al Suyyagh K, Dunkow P (2021). Financial impact of inaccurate coding plus cost-effectiveness analysis for surgically managed patients with periprosthetic fractures. Cureus.

[16] O'Malley KJ, Cook KF, Price MD, Wildes KR, Hurdle JF, Ashton CM (2005). Measuring diagnoses: ICD code accuracy. Health Serv Res.

[17] Kaur R (2019). Distributed knowledge based clinical auto-coding system. Proceedings of the 57th Annual Meeting of the Association for Computational Linguistics: Student Research Workshop.

[18] Donaldson MS, Corrigan JM, Kohn LT (2000). To Err is Human: Building a Safer Health System.

[19] Yeow JA, Ng PK, Tan KS, Chin TS, Lim WY (2014). Effects of stress, repetition, fatigue and work environment on human error in manufacturing industries. J Appl Sci.

[20] (2004). International statistical classification of diseases and related health problems: alphabetical index. World Health Organization.

[21] Chang NW, Dai HJ, Jonnagaddala J, Chen CW, Tsai RT, Hsu WL (2015). A context-aware approach for progression tracking of medical concepts in electronic medical records. J Biomed Inform.

[22] Yang Z, Yang D, Dyer C, He X, Smola A, Hovy E (2016). Hierarchical attention networks for document classificatio. Proceedings of the 2016 Conference of the North American Chapter of the Association for Computational Linguistics: Human Language Technologies.

[23] Radford A, Wu J, Child R, Luan D, Amodei D, Sutskever I (2019). Language models are unsupervised multitask learners. OpenAI blog.

[24] Dong H, Suárez-Paniagua V, Whiteley W, Wu H (2021). Explainable automated coding of clinical notes using hierarchical label-wise attention networks and label embedding initialisation. J Biomed Inform.

[25] Teng Q, Liu Z, Song Y, Han K, Lu Y (2022). A survey on the interpretability of deep learning in medical diagnosis. Multimed Syst.

[26] Yang R, Tan TF, Lu W, Thirunavukarasu AJ, Ting DS, Liu N (2023). Large language models in health care: development, applications, and challenges. Health Care Sci.

[27] Pennington J, Socher R, Manning CD (2014). Glove: global vectors for word representation. Proceedings of the 2014 Conference on Empirical Methods in Natural Language Processing.

[28] Papanikolaou Y, Pierleoni A Dare: data augmented relation extraction with gpt-2. arXiv.

[29] Devlin J, Chang MW, Lee K, Toutanova K (2019). BERT: pre-training of deep bidirectional transformers for language understanding. Proceedings of the 2019 Conference of the North American Chapter of the Association for Computational Linguistics: Human Language Technologies.

[30] Chen PF, Wang SM, Liao WC, Kuo LC, Chen KC, Lin YC, Yang C, Chiu C, Chang S, Lai F (2021). Automatic ICD-10 coding and training system: deep neural network based on supervised learning. JMIR Med Inform.

[31] Lai CH, Shian BT, Ke CR (2024). Demonstration website for the developed NLP-driven AI-assisted ICD-10-CM coding system. ISLAB.

[32] Kingma DP, Ba J Adam: a method for stochastic optimization. arXiv.

[33] Loshchilov I, Hutter F Decoupled weight decay regularization. arXiv.

[34] Landis JR, Koch GG (1977). The measurement of observer agreement for categorical data. Biometrics.

[35] Ji S, Li X, Sun W, Dong H, Taalas A, Zhang Y, Wu H, Pitkänen E, Marttinen P (2024). A unified review of deep learning for automated medical coding. ACM Comput Surv.

[36] Vu T, Nguyen DQ, Nguyen A (2020). A label attention model for ICD coding from clinical text. Proceedings of the 29th International Conference on International Joint Conferences on Artificial Intelligence.

[37] Wu Y, Zeng M, Yu Y, Li Y, Li M (2022). A pseudo label-wise attention network for automatic ICD coding. IEEE J Biomed Health Inform.

[38] Li M, Fei Z, Zeng M, Wu FX, Li Y, Pan Y, Wang J (2019). Automated ICD-9 coding via a deep learning approach. IEEE/ACM Trans Comput Biol Bioinform.

[39] Le Q, Mikolov T (2014). Distributed representations of sentences and documents. Proceedings of the 31st International Conference on International Conference on Machine Learning.

[40] Zeng M, Li M, Fei Z, Yu Y, Pan Y, Wang J (2019). Automatic ICD-9 coding via deep transfer learning. Neurocomputing.

[41] Tsatsaronis G, Balikas G, Malakasiotis P, Partalas I, Zschunke M, Alvers MR, Weissenborn D, Krithara A, Petridis S, Polychronopoulos D, Almirantis Y, Pavlopoulos J, Baskiotis N, Gallinari P, Artiéres T, Ngomo AN, Heino N, Gaussier E, Barrio-Alvers L, Schroeder M, Androutsopoulos I, Paliouras G (2015). An overview of the BIOASQ large-scale biomedical semantic indexing and question answering competition. BMC Bioinformatics.

[42] Bhutto SR, Zeng M, Niu K, Khoso S, Umar M, Lalley G, Li M (2024). Automatic ICD-10-CM coding via Lambda-Scaled attention based deep learning model. Methods.

[43] Zafirah SA, Nur AM, Puteh SE, Aljunid SM (2018). Potential loss of revenue due to errors in clinical coding during the implementation of the Malaysia diagnosis related group (MY-DRG) Casemix system in a teaching hospital in Malaysia. BMC Health Serv Res.

