# Supplementary material for: Evaluating a Natural Language Processing–Driven, AI-Assisted International Classification of Diseases, 10th Revision, Clinical Modification, Coding System for Diagnosis Related Groups in a Real Hospital Environment: Algorithm Development and Validation Study
Source: J Med Internet Res. 2024 Sep 20;26:e58278. doi: 10.2196/58278 (PMC11452756; doi:10.2196/58278)
Supplement: Multimedia Appendix 1 [file jmir_v26i1e58278_app1.docx]

**Appendix A. Supplementary Data for the Process of Tw-DRGs**


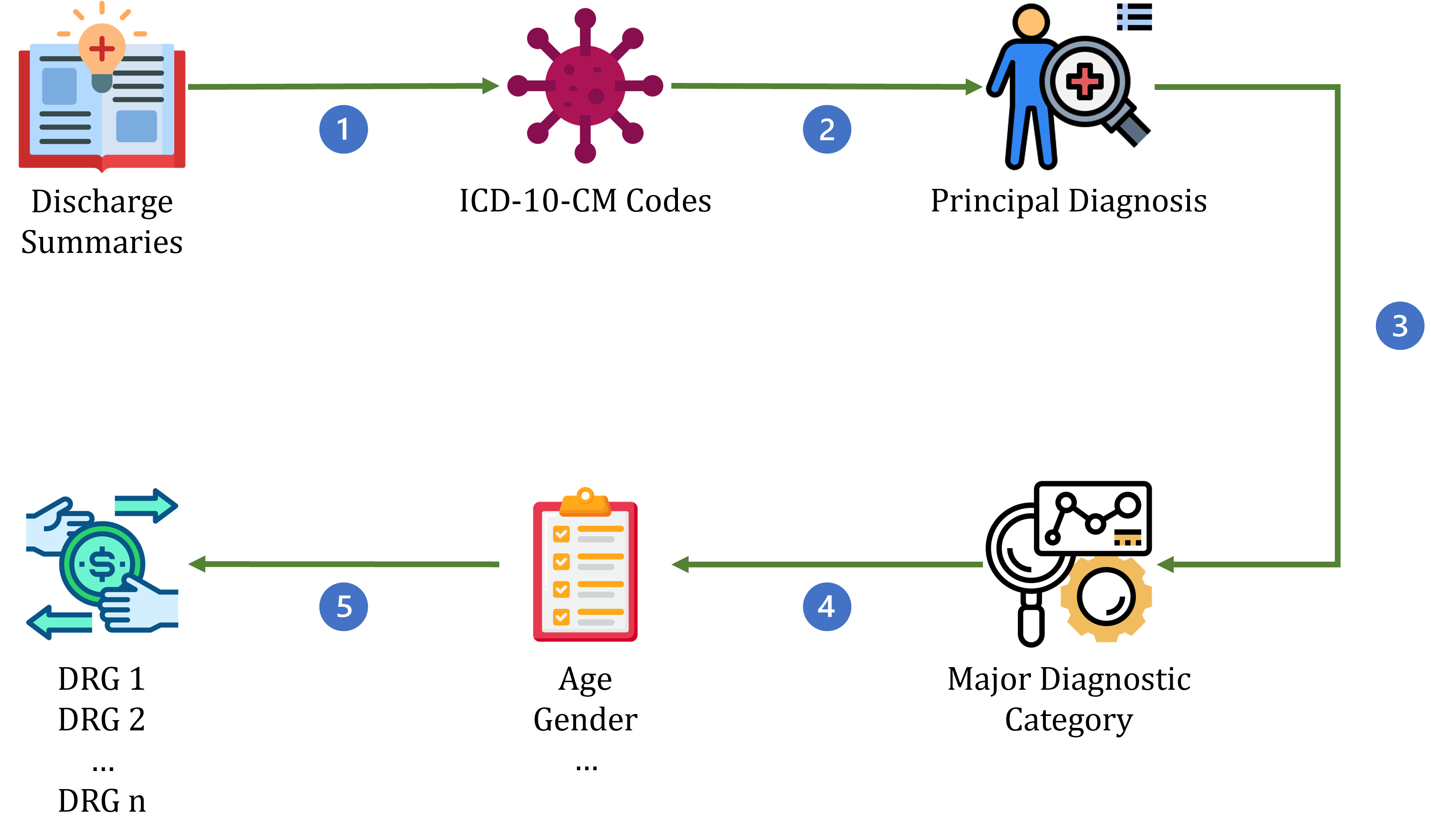


Figure S.1. The implementation process for Tw-DRGs.

Figure S.1 depicts the current implementation practice of the Taiwan Diagnosis Related Groups (Tw-DRGs) payment system. In the first and second steps of this process involves the conversion of diagnoses from physician-provided discharge summaries into ICD-10-CM codes and follows the principal diagnosis selection principle defined by National Health Insurance Administration (NHIA) to use the main cause of hospitalization as the principal diagnosis. In cases where multiple principal diagnoses are identified simultaneously, the one with the highest medical cost will be selected.

In the steps 3 to 5, the principal diagnosis representing the main cause of a patient’s hospitalization is subsequently categorized into the corresponding MDC under the execution of the Tw-DRG calculation software program, which considers various factors including the patient's age and gender, the presence of comorbidities or complications (secondary diagnoses), discharge status, etc. The MDC schematic classification for inpatient cases consists of 26 distinct categories, spanning from pre-MDC to MDC 1 through MDC 25. Once the principal diagnosis is determined for a patient, similar therapeutic diseases or procedures are further divided into multiple DRGs. The prospective inpatient costs that the NHIA should reimburse hospitals are calculated by leveraging historical data from the healthcare industry as a foundational reference.
