# Supplementary material for: Evaluating a Natural Language Processing–Driven, AI-Assisted International Classification of Diseases, 10th Revision, Clinical Modification, Coding System for Diagnosis Related Groups in a Real Hospital Environment: Algorithm Development and Validation Study
Source: J Med Internet Res. 2024 Sep 20;26:e58278. doi: 10.2196/58278 (PMC11452756; doi:10.2196/58278)
Supplement: Multimedia Appendix 2 [file jmir_v26i1e58278_app2.docx]

**Appendix B. Supplementary Data for the Top 50 ICD-10-CM Codes**

The following list presents the top 50 ICD-10-CM codes observed in the training set, sorted by their frequencies in descending order.

1. I10
2. Z5111
3. E119
4. E785
5. A419
6. N390
7. J189
8. C787
9. E1122
10. E876
11. B1910
12. I2510
13. N400
14. E871
15. Z5112
16. K922
17. N186
18. C7951
19. I129
20. I509
21. N179
22. C220
23. B181
24. C7800
25. D649
26. I6930
27. C50912
28. C50911
29. C20
30. K219
31. I120
32. D62
33. K7460
34. E860
35. C187
36. N189
37. K7290
38. K259
39. N183
40. D500
41. K210
42. I4891
43. K659
44. M109
45. B1920
46. Z992
47. D509
48. Z955
49. F329
50. R6521
